# Recapitulation of anti-aging phenotypes by global overexpression of PTEN in mice

**DOI:** 10.1007/s11357-023-01025-8

**Published:** 2023-12-19

**Authors:** Mary Hager, Peter Chang, Michael Lee, Calvin M. Burns, S. Joseph Endicott, Richard A. Miller, Xinna Li

**Affiliations:** 1 College of Literature, Sciences, & the Arts, University of Michigan, Ann Arbor, MI 48109 USA; 2 Department of Pathology, University of Michigan School of Medicine, Room 3160, BSRB ,109 Zina Pitcher Place, Ann Arbor, MI 48109-2200 USA; 3 University of Michigan Geriatrics Center, Ann Arbor, MI 48109 USA

**Keywords:** Slow-aging mice, Aging, Macrophage, Adipose tissue, Hippocampus, Liver, Phosphatase and tensin homolog (PTEN), Uncoupling protein 1 (UCP1), Fibronectin type III domain-containing protein 5 (FNDC5)/IRISIN, Glycosylphosphatidylinositol-specific phospholipase D1 (GPLD1), Brain-derived neurotrophic factor (BDNF), Doublecortin (DCX))

## Abstract

The PTEN gene negatively regulates the oncogenic PI3K-AKT pathway by encoding a lipid and protein phosphatase that dephosphorylates lipid phosphatidylinositol-3,4,5-triphosphate (PIP_3_) resulting in the inhibition of PI3K and downstream inhibition of AKT. Overexpression of PTEN in mice leads to a longer lifespan compared to control littermates, although the mechanism is unknown. Here, we provide evidence that young adult PTENOE mice exhibit many characteristics shared by other slow-aging mouse models, including those with mutations that affect GH/IGF1 pathways, calorie-restricted mice, and mice treated with anti-aging drugs. PTENOE white adipose tissue (WAT) has increased UCP1, a protein linked to increased thermogenesis. WAT of PTENOE mice also shows a change in polarization of fat-associated macrophages, with elevated levels of arginase 1 (Arg1, characteristic of M2 macrophages) and decreased production of inducible nitric oxide synthase (iNOS, characteristic of M1 macrophages). Muscle and hippocampus showed increased expression of the myokine FNDC5, and higher levels of its cleavage product irisin in plasma, which has been linked to increased conversion of WAT to more thermogenic beige/brown adipose tissue. PTENOE mice also have an increase, in plasma and liver, of GPLD1, which is known to improve cognition in mice. Hippocampus of the PTENOE mice has elevation of both BDNF and DCX, indices of brain resilience and neurogenesis. These changes in fat, macrophages, liver, muscle, hippocampus, and plasma may be considered “aging rate indicators” in that they seem to be consistently changed across many of the long-lived mouse models and may help to extend lifespan by delaying many forms of late-life illness. Our new findings show that PTENOE mice can be added to the group of long-lived mice that share this multi-tissue suite of biochemical characteristics.

**Supplementary Information:**

The online version contains supplementary material available at 10.1007/s11357-023-01025-8.

## Introduction

Phosphatase and tensin homolog deleted on chromosome 10 (PTEN, also known as MMAC1 and TEP1) is a highly studied tumor-suppressor gene whose mutated form is commonly linked to oncogenesis [1, 2]. PTEN contains a tensin-like domain and a phosphatase catalytic domain and is produced in all tissues [3]. PTEN negatively regulates the INS/PI3K/AKT pathway by encoding a lipid and protein phosphatase that dephosphorylates PI (3,4,5) P_3_, thereby inhibiting downstream activation of AKT [4]. PTEN is known to upregulate UCP1 expression in brown adipocytes, which enhances their nutrient-burning thermogenic capacity [5]. PTEN over-expressing transgenic mice (PTENOE mice) exhibit increased metabolism, decreased adiposity, decreased insulin resistance in the context of high-fat feeding or aging, and longer lifespan [6, 7].

Uncoupling protein 1 (UCP1) resides in the mitochondrial membrane of brown adipocytes and is a major contributor to adaptive thermogenesis [8]. UCP1 transports protons across the inner membrane of mitochondria resulting in the “uncoupling” of cellular respiration from ATP synthesis. This, in turn, releases energy in the form of heat while also stimulating fatty acid oxidation [9, 10]. Due to this process, BAT is a major source of heat production in mammals. In addition, WAT can be “activated” by certain stimuli. In response to cold, the body produces β-adrenergic agonists including norepinephrine, which in turn results in the production of UCP1-expressing adipocytes in white adipose tissue (WAT) [11–13]. The newly thermogenic white adipocytes are re-categorized as beige, “brite” (brown in white), iBAT (induced BAT), recruitable BAT, and wBAT (white adipose BAT) cells [14]. These beige cells have thermogenic abilities similar to brown adipocytes [15], but differ from BAT in other respects [16, 17].

Adipose tissue is crucial for health, both for energy storage and as an important endocrine organ [18, 19]. Macrophages that reside within adipose tissue play a large role not only in immune health but also in the secretion of cytokines that affect metabolism [20, 21]. Macrophages have many different activation states that change in response to microenvironmental factors [21]. A useful categorization focuses on macrophage polarization states M1 and M2, each with different and often opposing properties. While cells with intermediate phenotypes exist, the classically activated M1 macrophage promotes an inflammatory environment, and the alternatively activated M2 cells reduce inflammatory change [22–24]. M1 macrophages secrete pro-inflammatory cytokines and chemokines, such as TNF-α, interleukin IL-6, and MCP-1. M1 cell function is mainly anti-bacterial and apoptotic. In contrast, M2 cells are anti-inflammatory and anthelminthic and promote wound healing through tissue regeneration through the secretion of arginase-1, IL-10, IL-4, and other cytokines [21, 25–27]. M2 macrophages have also been found to increase UCP1 levels in adipocytes and promote the browning of WAT [28]. Aging is often accompanied by chronic low-grade inflammation and the metabolic concomitants of obesity [29–31]. Adipose tissue inflammation is characterized by an increase in adipose tissue macrophages and a change in polarization from M2 to M1 which coincides with increased insulin resistance [27]. As a result, the M1/M2 ratio can be used as an index of this age-related inflammation [32, 33].

FNDC5 [34, 35] is an exercise-induced myokine that is found in many tissues including the heart, brain, ovary, testis, kidney, stomach, liver, and mainly skeletal muscle [36]. It undergoes post-translational processing to generate the plasma protein irisin. Irisin plays a role in WAT browning by increasing UCP1 expression [37]. In adipose tissue, it has been recently discovered that irisin plays a role in the conversion of the macrophage populations from inflammatory M1 to anti-inflammatory M2 [38] resulting in the reduction of pro-inflammatory cytokines (TNFα, IL-1β, IL-6, MCP-1) and the increase of anti-inflammatory cytokines (IL-10, IL-4, IL-13) [37–39]. FNDC5/Irisin were shown to mediate the benefits of exercise on cognitive ability [39], perhaps through the upregulation of BDNF expression [40–42]. More recent literature has supported the hypothesis that FNDC5 and irisin have a necessary role in exercise-related benefits in the aging body [43].

Loss of memory is a common symptom of aging [44]. Brain-derived neurotrophic factor (BDNF) is a key molecule involved in the maintenance of brain plasticity [45]. The loss of BDNF expression has been linked to hippocampal dysfunction as a result of age, impaired memory, and increased depression risk [46]. In addition to BDNF, the expression of microtubule-associated protein doublecortin (DCX) [47] has a positive relationship with brain health in aging [47–49]. DCX is often used as an index for neurogenesis due to its exclusive presence in developing neurons [50–52].

Horowitz et al. have documented induction of the hepatic protein GPLD1 (glycosylphosphatidylinositol (GPI)-degrading enzyme) by exercise and its resulting secretion into the bloodstream and showed further that overexpression of GPLD1 in the liver of aged mice resulted in improvements in cognitive ability within 3 weeks of treatment [53]. This correlation was supported by evidence of increased levels of BDNF and DCX in the hippocampus. Interestingly, it was discovered that GPLD1 does not cross the blood brain barrier [53]. Thus, the mechanism behind GPLD1’s regulation of brain function is not fully understood but may involve pathways that reduce inflammation and blood coagulation throughout the body [53]. Work in our own laboratory showed elevation of liver GPLD1 protein in many varieties of slow-aging mice [54–57] and showed that the elevation of GPLD1 was not accompanied by a change in mRNA level, but instead reflects differential mRNA translation via cap-independent translation (CIT) [55]. Previous work had documented an increase in CIT in long-lived mutant mice (Snell, Ames, GHRKO), and the identification of GPLD1 as a CIT protein provides a link between translational regulation and modulation of brain function in these long-lived mice [58, 59].

Thus, recent work has identified a set of characteristics (“aging rate indicators,” or ARI) shared in adult mice that have been exposed to a wide range of genetic, dietary, or pharmacological anti-aging interventions. The interventions include mutations (Ames, Snell, GHRKO, and PAPPA-KO), CR diet, or mice treated with drugs such as rapamycin (Rapa), acarbose (Aca), 17aE2, or canagliflozin (Cana) [54–57]. These models share physiological changes including increases in uncoupling protein UCP1 in brown and white adipose tissue (WAT); a change in fat-associated macrophage subsets that leads to diminished production of inflammatory cytokines; an increase in muscle FNDC5 and plasma irisin, elevated production of GPLD1 and in liver and plasma, and elevation of hippocampal BDNF and DCX. Drugs whose effect on lifespan is specific for males modulate some of the ARIs in males but not in females [57]. Interference with a genetic effect on lifespan, specifically by short-term early-life exposure of Ames dwarf mice to growth hormone, also blocks the changes in the ARI seen in mock-treated Ames mutants [54]. Taken together, the correlation between unusually long lifespan and changes in the collection of ARIs suggests the hypothesis that other varieties of long-lived mice might show corresponding changes in ARIs. We therefore tested whether PTENOE mice also display alterations of ARIs in multiple tissues, similar to those seen in other varieties of slow-aging mice. Our new results support this idea and provide new insights into the pathways through which PTEN affects metabolism, cancer, obesity, diabetes, and aging.

## Materials and methods

### Mice

A female mouse on an inbred background of approximately 75% C57BL/6 and 25% CBA, carrying a single copy of the *pten* transgene, was a gift from Daniel Herranz at Rutgers University. This female was crossed to a BALB/cByJ (JAX stock 0012026) male. The seven subsequent generations were produced by breeding males heterozygous for the *pten* transgene to CByB6F1/J females (JAX stock 100009), which are F1 hybrids from BALB/cByJ mothers and C57BL/6 J fathers. Thus, the *pten* transgene was crossed onto a segregating background of 50% C57BL/6 J and 50% BALB/cByJ. All experimental animals carried a single copy of the *pten* transgene, with transgene-negative littermates used as controls. All experimental animals are young adult (4–6 months old) mice of both sexes.

### Genotyping of mice and tissues

To identify the presence of the *pten* transgene, DNA was isolated from ear notches and subjected to PCR using primers: 5′-CCGCTAATACGACTCACTATAGGG-3′ (forward) and 5′-TCATCTCGGCTCCATCGTTT-3′ (reverse). The PCR protocol was 94 °C for 3 min, followed by 35 cycles of 94 °C for 30 s, 60 °C for 30 s, and 72 °C for 30 s, with a final extension of 72 °C for 3 min, and a hold at 4 °C. This protocol produces an approximately 200 bp product, indicating the presence of the *pten* transgene.

### RNA isolation and cDNA synthesis

Tissue samples were taken from young adult (4–6 months old) mice of both sexes. Samples were homogenized utilizing the Blender PRO250 (Pro Scientific Inc, CT, USA). Total RNA was isolated from mouse livers and adipose tissues using Trizol kit (Cat # 19424, Sigma-Aldrich, Inc, St. Louis, MO) according to the manufacturer’s instruction. The RNA was cleaned using the QiagenRNeasy miniRNA cleanup kit (Cat# 74204, Qiagen, Valencia, CA). The concentration of total RNA was performed by measuring the absorbance of RNA sample solutions at 260 nm by using a Nanodrop ND-100. Total RNA (1.0 μg) was reverse transcribed using iScript cDNA reverse transcription kits (Cat# 1708891; Bio-Rad, Hercules, CA) according to the manufacturer’s instructions.

### Quantitative real-time PCR

qPCR was performed using the Fast Start Universal SYBR Green Master Mix (Applied Biosystems, Foster City, CA). RT-PCR was performed using quantitative PCR systems (Applied Biosystems® 7500 Real-Time PCR Systems, Thermo Fisher Scientific, Waltham, MA, USA) with corresponding primers (Table S1, Invitrogen). Glyceraldehyde-3-phosphate dehydrogenase (GAPDH) was simultaneously assayed as a loading control. The expression levels of mRNA were reported as fold changes vs. sham control. Reactions were performed using an Applied Biosystems 7500 Real-Time RT-PCR System. Data was analyzed using a ΔΔCT approach.

### Western blot analyses

Proteins from various tissues of the experimental mice (brown adipose, inguinal adipose, perigonadal adipose, liver, muscle, and hippocampus) were extracted after homogenization in Immunoprecipitation Assay Buffer (RIPA Buffer, Fisher Scientific, Pittsburgh, PA, USA) supplemented with Complete Protease Inhibitor Cocktail (Roche Inc.). Protein content was measured using a BCA assay (Fisher Scientific, Pittsburgh, PA, USA). The protein extracts were separated by SDS/PAGE on a 4–15% running gel, transferred to polyvinylidene difluoride membranes and electro-transferred to an Immobilon-P Transfer Membrane (Millipore, Billerica, MA, USA) for immune blot analyses. Membranes were blocked in Tris-buffered saline containing 0.05% Tween20 (TBS-T) and 5% bovine serum albumin (BSA) for 1 h. After blocking, membranes were probed overnight with primary antibodies in TBS-T supplemented with 5% BSA with shaking at 4 °C, followed by three 10-min washes with TBS-T, incubation with secondary antibody for 1 h, and three 10-min washes with TBS-T. Membranes were then evaluated using an ECL Chemiluminescent Substrate (Fisher Scientific, Pittsburgh, PA, USA). The following antibodies were used: anti-GPLD1 (Abcam, catalog no. 210753, 1:1000), anti-BDNF (Abcam, catalog no. 108319, 1:1000), anti-Doublecortin (Abcam, catalog no. 18723, 1:1000), anti-β-actin (Santa Cruz Biotechnology, 1:1000), HRP-conjugated anti-mouse (GE Healthcare UK Limited, 1:2000), and anti-rabbit (GE Healthcare UK Limited, 1:5000). Quantification was performed using ImageJ software. Table S2 provides a list of the antibodies used.

### Measurement of IGF-1, irisin, and GPLD1 levels by enzyme-linked immunosorbent assay

Blood samples from the PTENOE mice were collected into ethylene diamine tetraacetic acid (EDTA)–coated tubes, and plasma was isolated by centrifugation (10,000 rcf, 4˚C, 10 min). ELISA kits were used to determine the levels of IGF-1 (Cat# LS-F5608, LSBio, Shirley, MA), FNDC5/irisin (Cat# LS-F23848, LSBio, Shirley, MA) and GPLD1 irisin (Cat# LS-F17042, LSBio, Shirley, MA) according to the standard protocol. Briefly, serum samples from PTENOE mice were added into each well and incubated at room temperature for 120 min. Then, the biotin-conjugated antibody was added, followed by incubation for 90 min. Thereafter, the substrate solution was added to the well and incubated for 30 min. After washing five times with 0.01 mol/L tris-buffered saline (TBS), 3,3′,5,5′-tetramethylbenzidine (TMB) was added and incubated for 30 min in the dark. The absorbance at 450 nm was determined using a Bio-Rad iMark microplate reader.

### Statistical analysis

The data shown in each figure represent results of a minimum of three independent experiments. All data are presented as mean ± SEM. A two-way ANOVA test, with sex, treatment, and interaction terms, was used for comparisons of experimental groups. *p* < 0.05 was regarded as significant.

## Results

### PTEN regulates body weight and body size

**Fig. 1:**
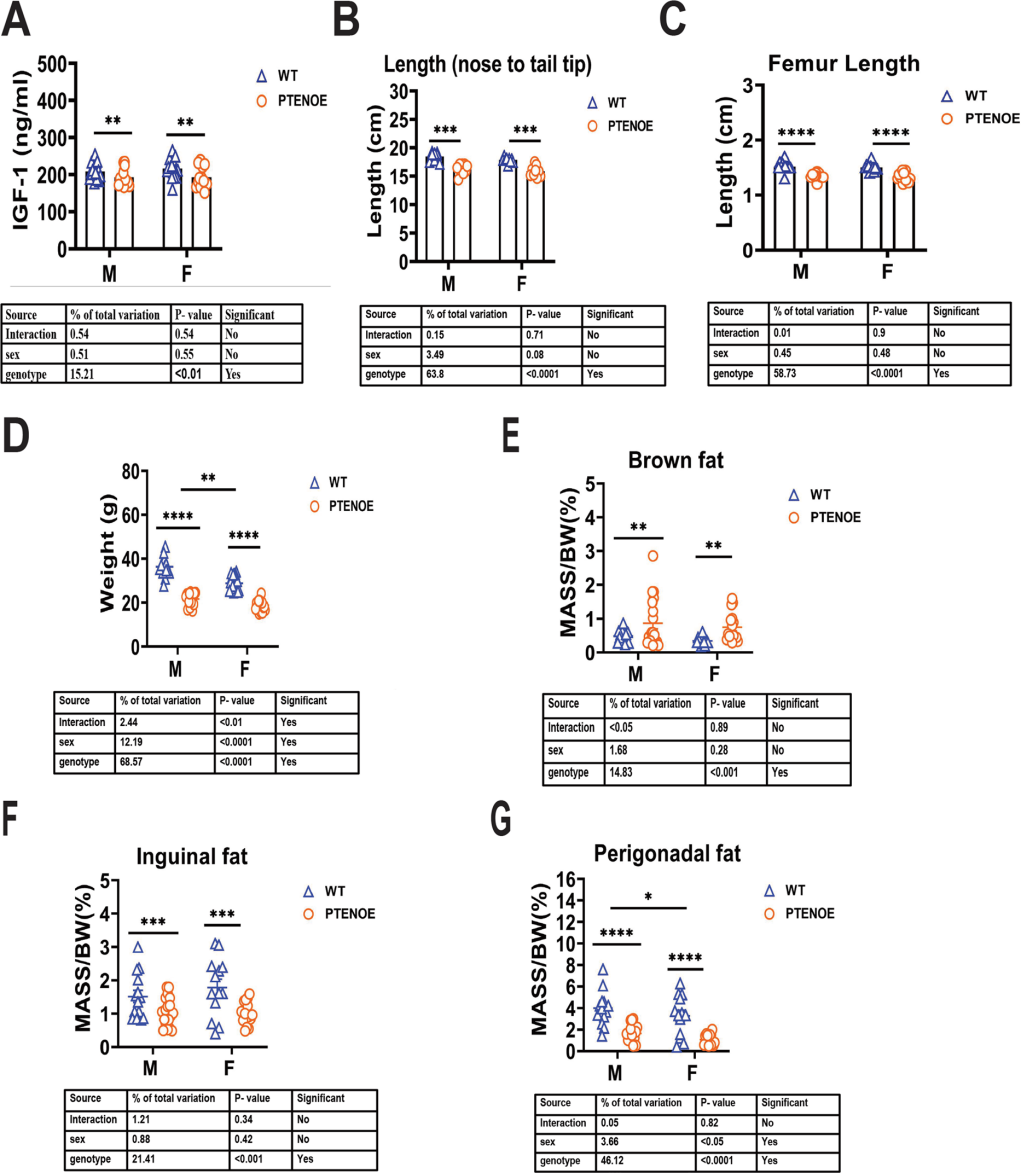
Effects of PTEN on levels of IGF-1 in plasma, body length, body mass, and adipose tissue mass. **A** IGF-1 protein was measured by ELISA assay on plasma samples of 24-week-old wild-type littermate control mice (WT) and PTENOE mice (*n* = 13–18 per group). In each panel, data are shown as mean ± SEM. ****p < 0.0001 by two-way ANOVA. **B**, **C** Body length (from nose to tail tip) and femur length were measured in 16-week-old mice. (*n* = 15–19). **E**, **F**, **G** Mass of BAT, ING WAT, and PG WAT in 16-week-old mice (*n* = 15–19). Asterisks in panels without interaction effect indicate significance for genotype or sex effect in two-way ANOVA: ****p < 0.0001; ***p < 0.001; **p < 0.01, * *p* < 0.05. Asterisks in **D** reflect *t*-tests done separately in each sex

In our study, we identified that PTEN protein level increased in various tissues, such as liver, muscle, hippocampus, brown fat, and inguinal and perigonadal fat of PTENOE mice (Supplementary Fig. 1 & 2). We noted that PTENOE mice had lower plasma IGF1, body length (nose to tail tip), femur length, and body weight than littermate controls (Fig. 1A–D). Both sexes were affected to a similar degree, except for the data on body weight, where the relative decline was greater in males than in females. As shown in Fig. 1D, PTEN overexpression resulted in 44% weight reduction for male and 40% for females. Reductions in body weight of PTENOE have been reported previously [3]. It is likely that the lower levels of IGF1 underlie the lower femur and overall body length, but we do not have measures of IGF1 at pertinent juvenile ages to test this idea. PTENOE mice had more BAT (as a percentage of body weight) than controls, but lower percentages of inguinal (ING) and perigonadal (PG) fat mass (Fig. 1E–G). Males had more PG fat than females, but there were no sex effects on BAT or ING fat, and no significant sex differences in the effects of PTEN.

### UCP1 protein levels in BAT and WAT of PTENOE mice

**Fig. 2:**
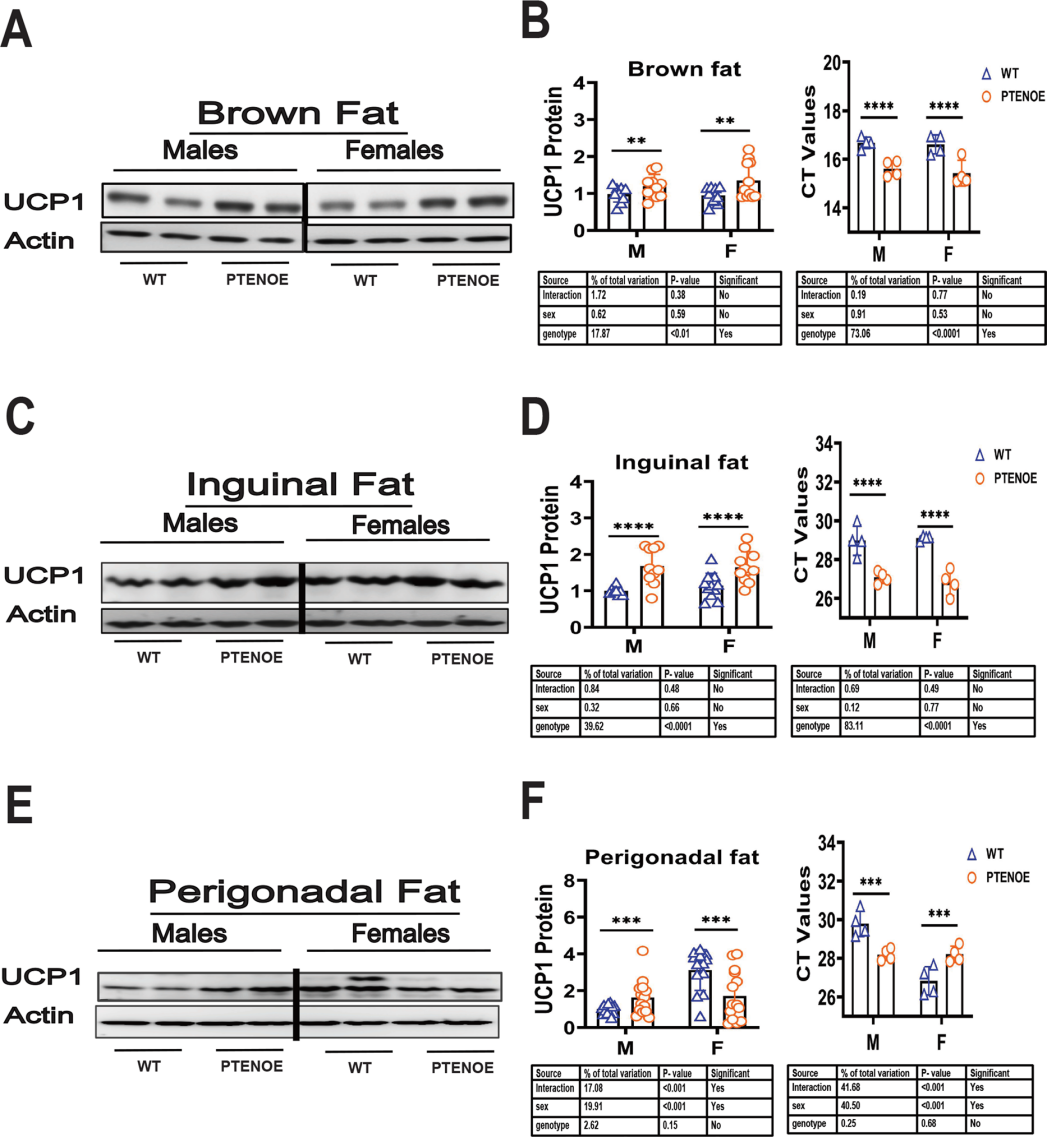
Expression of UCP1 in adipose tissue of PTENOE mice. **A**, **C**, **E** Cell lysates were prepared from adipose tissues of 16-week-old WT and PTENOE mice (*n* = 9–15). Protein levels of UCP1 (brown and beige fat marker) were measured by western blotting. Representative gel images showing UCP1 in brown (**A**), inguinal (**C**), and perigonadal adipose tissue (**E**). **B**, **D**, **F** Protein quantification data normalized to β-actin and expressed as fold change compared with WT control (defined as 1.0). Data are means ± SEM. **p < 0.01; ***p < 0.001; ****p < 0.0001 versus WT

Our previous work has shown increased levels of UCP1 in BAT, ING WAT, and PG WAT fat depots of nine slow-aging mice models (Snell dwarf, Ames dwarf, GHRKO, and PAPPA-KO mice and CR diet, and drug treatments) [54–57]. To see if similar changes were induced by the overexpression of PTEN, we evaluated fat tissues from 4- to 5-month-old PTENOE. Figure 2 shows representative immunoblot images, with dotplots to show the distribution of densitometric results. As shown in Fig. 2A and C, UCP1 protein levels are higher in BAT and ING WAT of PTENOE mice compared to wild-type littermate controls, and the non-significant interaction term of the two-factor ANOVA suggests that both sexes are equally affected by the genotype effect. PTENOE effects on UCP1 mRNA (Fig. 2B, D) are consistent with the protein changes. In contrast, PG WAT shows increased UCP1 protein only in males, while females show a significant decline in the PTENOE group (Fig. 2E, F). UCP1 mRNA data for PG WAT are consistent, and the ANOVA shows a significant [sex × genotype] term for both protein and mRNA. Results of these two-factor ANOVA calculations, for each endpoint used in this report, are collected in Table S3.

### Shift in macrophage subsets from inflammatory to anti-inflammatory status

**Fig. 3:**
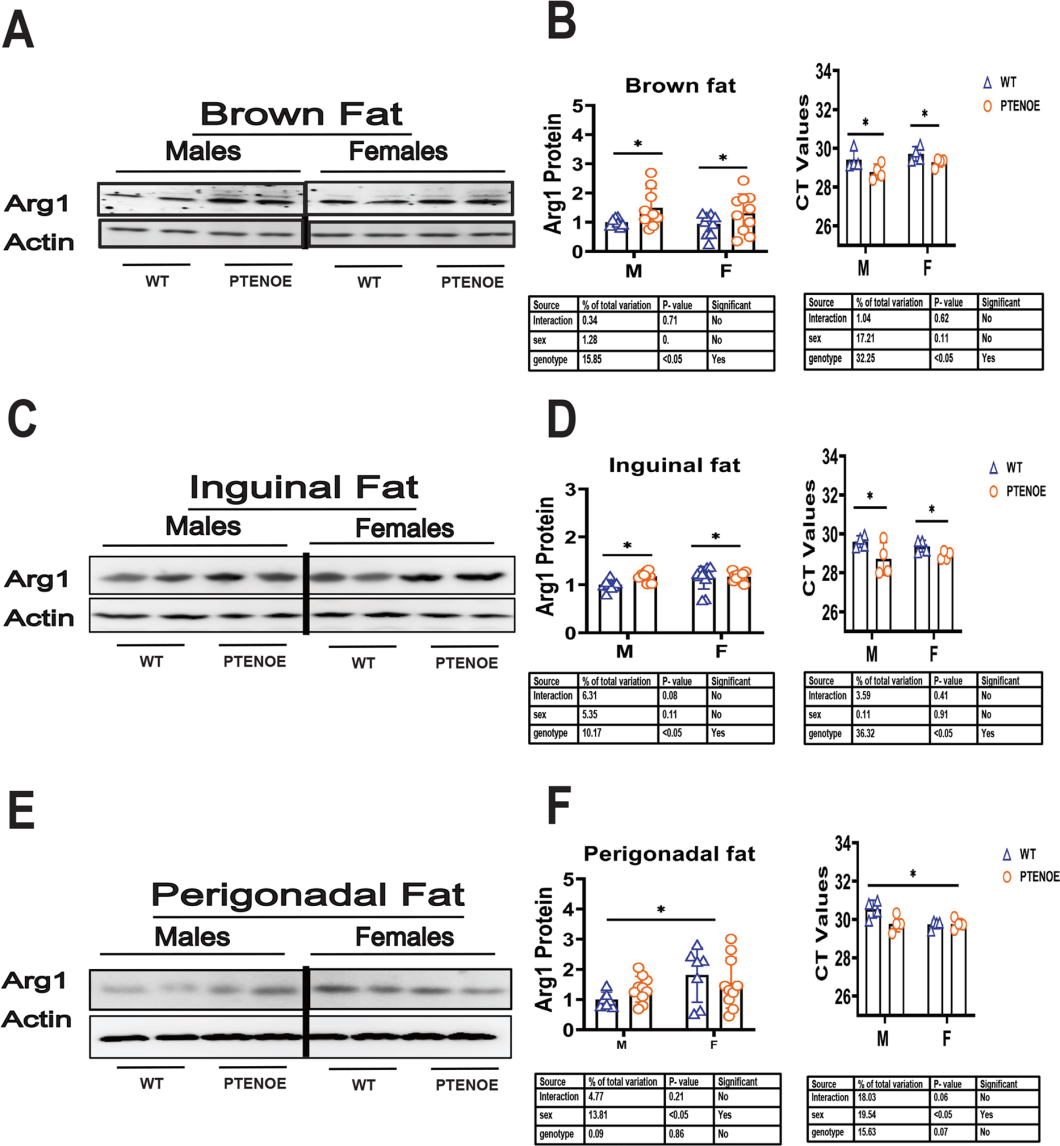
Expression of M2 macrophage marker Arg1 in adipose tissue of PTENOE mice. **A**, **C**, **E** Cell lysates were prepared from adipose tissues of 16-week-old wild-type littermate control mice (WT) and PTENOE mice (*n* = 7–10). Protein levels of Arg1 (M2 macrophage marker) were then measured by western blotting. Representative gel images showing Arg1 in brown (**A**), inguinal (**C**), perigonadal adipose tissue (**E**). **B**, **D**, **F** Protein quantification data normalized to β-actin and expressed as fold change compared with WT control (defined as 1.0). Data are means ± SEM. *p < 0.05 versus WT

**Fig. 4:**
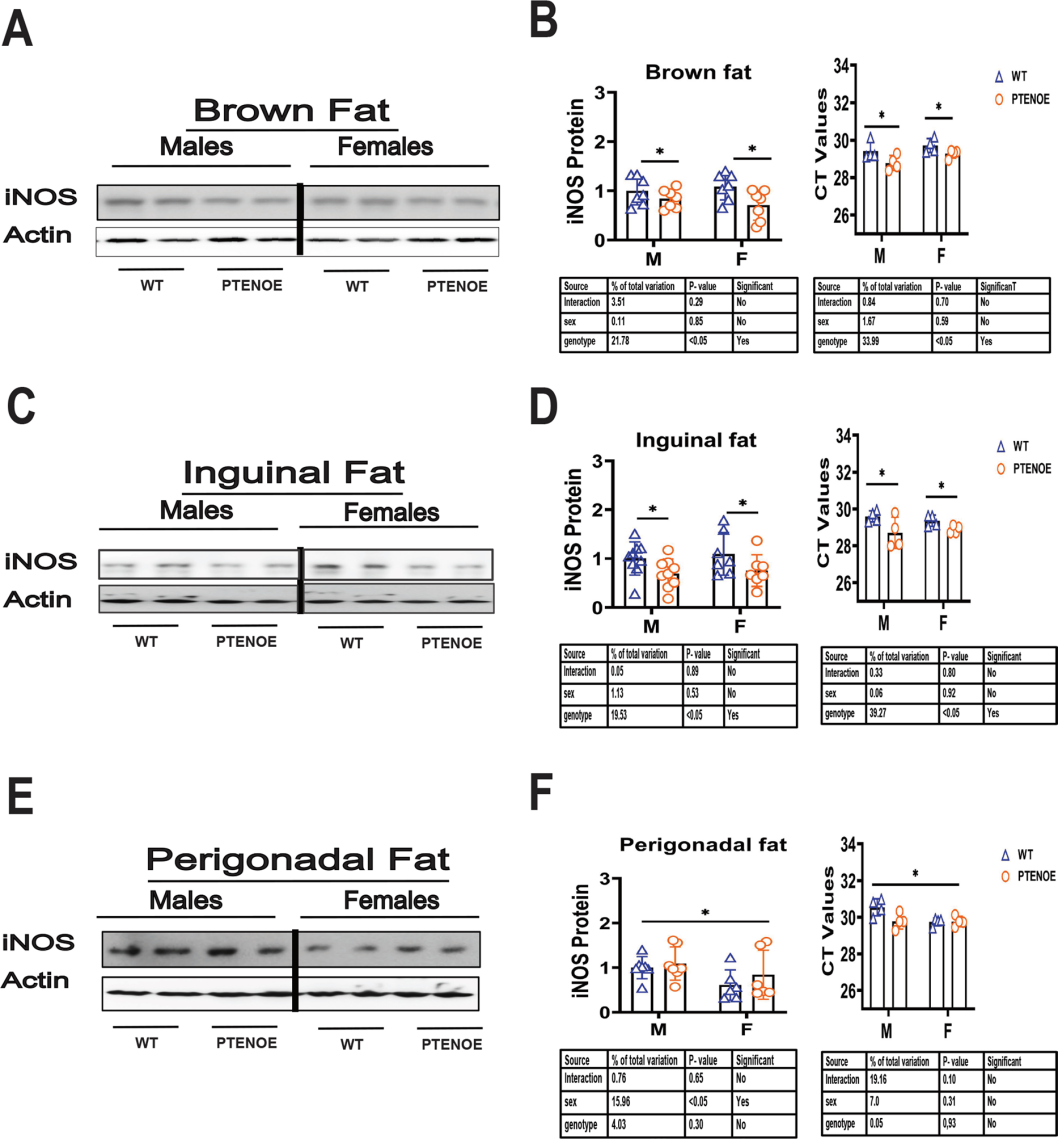
Expression of M1 macrophage marker in adipose tissue of PTENOE mice. **A**, **C**, **E** Cell lysates were prepared from adipose tissues of 16-week-old wild-type littermate control mice (WT) and PTENOE mice (*n* = 7–10). Protein levels of iNOS (M1 macrophage marker) were then measured by western blotting. Representative gel images showing iNOS in brown adipose tissue (**A**), inguinal adipose tissue (**C**), and perigonadal adipose tissue (**E**). **B**, **D**, **F** Protein quantification data normalized to β-actin and expressed as fold change compared with WT control (defined as 1.0). Data are means ± SEM. *p < 0.05 versus WT

We used Arg1 (Fig. 3) as an index of M2 macrophages, and iNOS (Fig. 4) as an index of M1 inflammatory macrophages, chosen from a broader panel of molecular and histologic markers employed in our previous work on changes in fat-associated macrophage subsets in slow-aging mice [54–57]. Our previous studies of long-lived mutant mice showed increases in M2 macrophages and declines in the M1 macrophage subset in BAT and WAT [54–57]. In our current study of PTENOE mice, Arg1 was found to be significantly elevated in BAT, and in ING WAT of PTENOE mice (Fig. 3A, C) by factors of 1.2- to 1.4-fold. In contrast, PTENOE did not alter Arg1 levels in PG WAT, although there was a significant sex effect, with Arg1 higher in females than in males regardless of genotype. Each of these results on Arg1 protein was consistent with parallel measures of Arg1 mRNA (Fig. 3B, D, E).

The data on iNOS (Fig. 4), an index of M1 macrophages, mirror those for Arg1 in BAT and ING WAT. iNOS is diminished significantly in BAT, and in ING WAT of PTENOE mice (Fig. 4A, C), to levels 60–70% of those in control mice; see Table S3. In contrast, levels of iNOS protein in PG WAT show no effect of PTENOE genotype, although females show diminished iNOS, consistent with the elevation of Arg1 protein in PG WAT (Fig. 3). Data on iNOS mRNA are consistent with the PTENOE effects on iNOS protein in BAT and ING WAT, and with the lack of genotype effect on PG WAT, suggesting an important role for transcriptional control of the expression of these proteins.

### Muscle FNDC5 and plasma irisin changes consistent with the alterations of adipose tissue

**Fig. 5:**
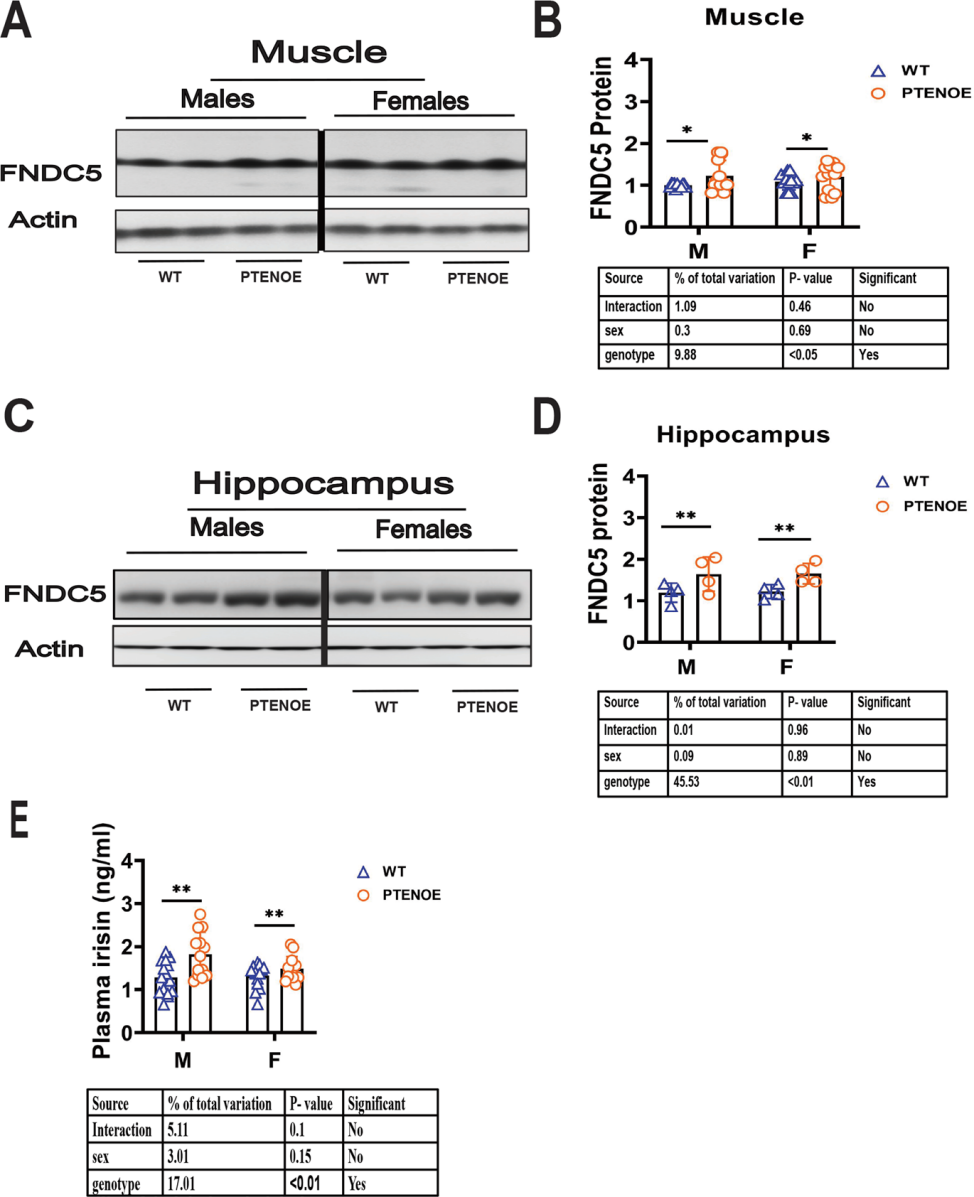
Expression of FNDC5 in gastrocnemius muscle and hippocampus and irisin levels in plasma of PTENOE mice. **A** Cell lysates were prepared from gastrocnemius muscle of 16-week-old wild-type littermate control mice (WT) and PTENOE mice (*n* = 7–10). Protein levels of FNDC5 were measured by western blotting. Representative gel images are shown. **B** Protein quantification data normalized to β-actin and expressed as fold change compared with WT control (defined as 1.0). Data are means ± SEM. **p < 0.01 versus WT. **C** Representative gel images showing FNDC5 in hippocampus. **D** Protein quantification data for hippocampus (*n* = 4–6). Data are means ± SEM. *p < 0.05; ** *p* < 0.01 versus WT. **E** Irisin measured by ELISA assay on plasma samples of 16-week-old wild-type littermate control mice (WT) and long-lived mice (PTENOE). Data are shown as mean ± SEM for each group (*n* = 13–15). *p < 0.05 for genotype effect by two-way ANOVA

In our previous studies, we found elevations of FNDC5 in muscle, and of its cleavage product, irisin, in plasma of slow aging mice [54–57, 60]. We therefore looked at FNDC5 and plasma irisin in the PTENOE mice in our current study. As shown in Fig. 5A, B, muscle FNDC5 protein was significantly elevated in PTENOE mice. Plasma irisin, the secreted portion of the FNDC5 molecule, was also elevated significantly in PTENOE mice compared to their littermate controls, significant at *p* < 0.05 (Fig. 5E). These data, along with previous results on slow aging mice [54–57, 60], support the idea that the changes in the fat depots of the various slow-aging mice might be secondary to increases in muscle FNDC5 and plasma irisin.

FNDC5 is highly expressed in the brain [34, 61, 62], in addition to muscle, and plays an important role in neuron development [62]. Recent data suggests that increased FNDC5 levels in hippocampus can improve cognitive function in mice [63]. We therefore evaluated FNDC5 in hippocampal tissues of PTENOE mice and noted a significant increase in PTENOE mice (1.4-fold higher, *p* < 0.01; Fig. 5C, D) that was seen in both sexes.

### GPLD1 levels in tissues and plasma of PTENOE mice

**Fig. 6:**
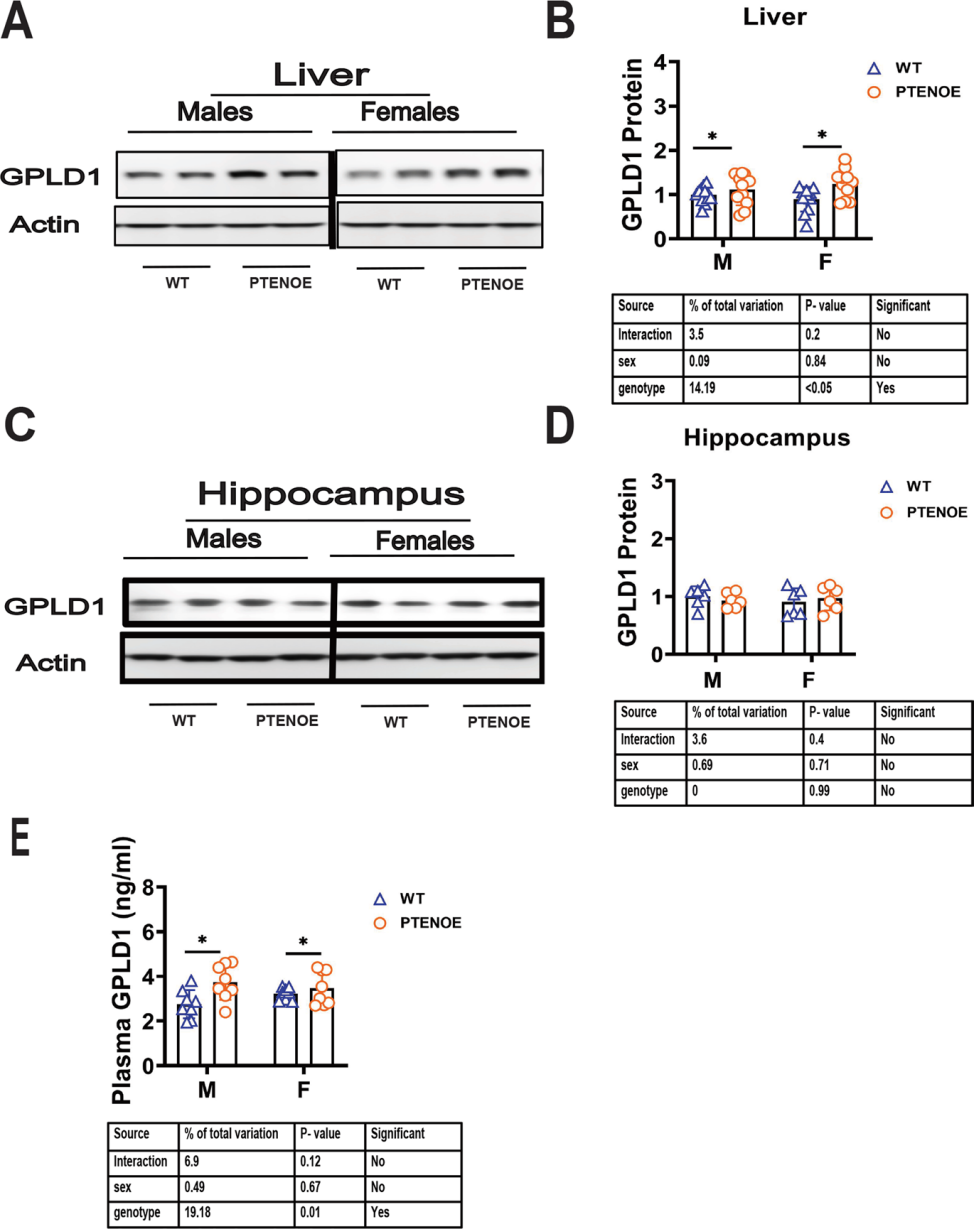
Effects of PTEN on GPLD1 in liver, hippocampus, and plasma. **A**, **C** Cell lysate was prepared from liver and hippocampus of 16-week-old wild-type littermate control mice (WT) and PTENOE mice. Protein levels of GPLD1 were then measured by western blotting. Representative gel images showing GPLD1 in liver tissue (*n* = 11) (**A**), hippocampus tissue (*n* = 6 per group) (**C**). **B**, **D** Protein quantification data normalized to β-actin and expressed as fold change compared with WT control (defined as 1.0). *p < 0.05 versus WT. **E** GPLD1 protein was measured by ELISA assay on plasma samples of 16-week-old mice. Data are shown as mean ± SEM for each group (*n* = 6). **p < 0.01 for genotype effect by two-way ANOVA

GPLD1 is elevated in liver and plasma of many other slow-aging mouse models [54–57]. Figure 6A and B show that liver GPLD1 protein is higher in PTENOE mice than in controls (1.3-fold higher, *p* < 0.05). Plasma levels of GPLD1 are also higher in PTENOE mice (Fig. 6E). GPLD1 protein can also be detected in hippocampus, but at levels that are not altered in long-lived Snell and GHRKO mice [54–57]. Consistent with the results in the other long-lived mutants [54–57], PTENOE mice do not show significant alterations in hippocampal GPLD1.

### BDNF and DCX elevation in hippocampus of PTENOE mice

**Fig. 7:**
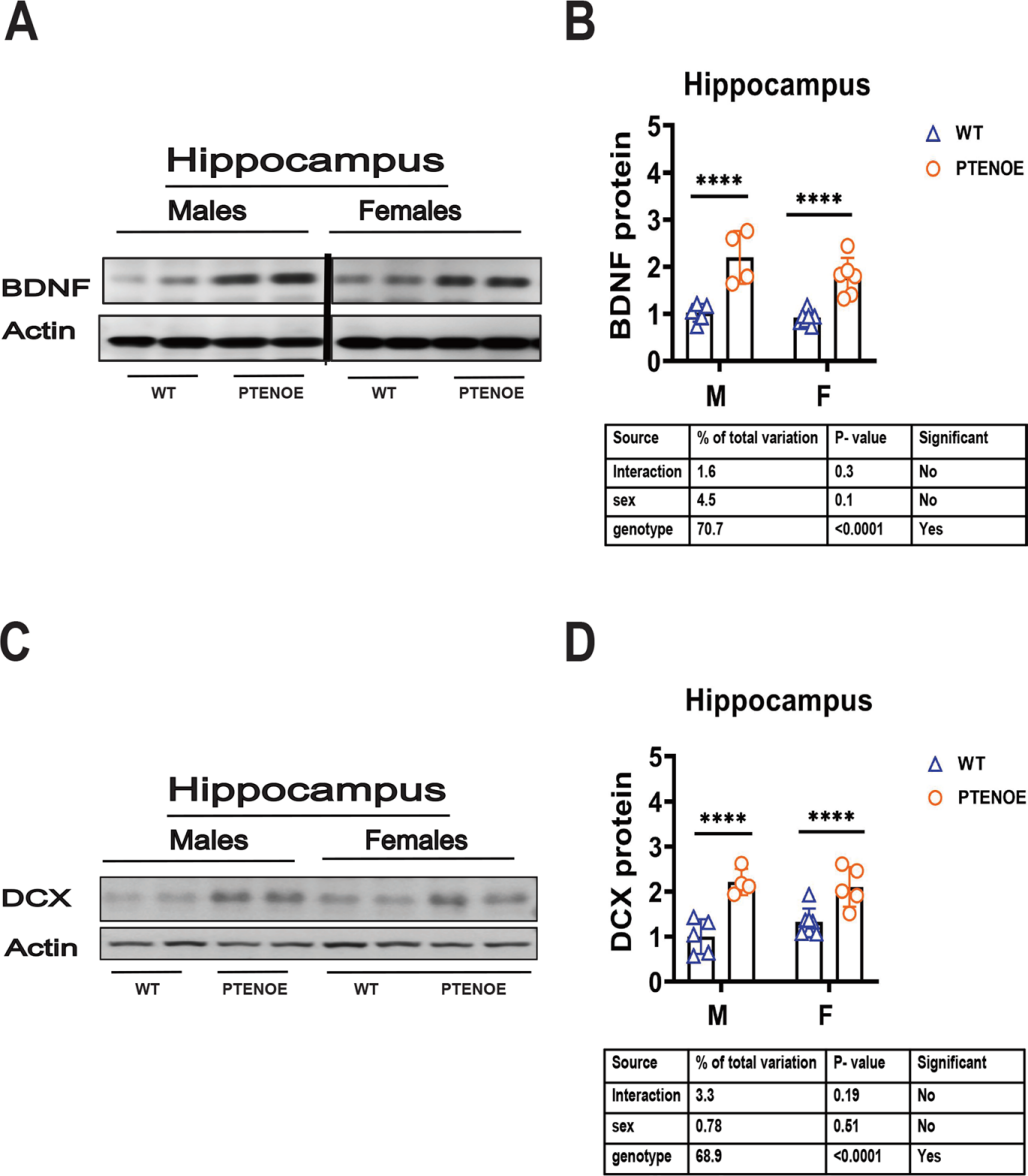
Expression of BDNF and doublecortin (DCX) in hippocampus of PTENOE mice. **A** Cell lysates were prepared from hippocampus of 16-week-old wild-type littermate control mice (WT) and PTENOE mice. Protein levels of BDNF were measured by western blotting. Representative gel images are shown. **B** Protein quantification data normalized to β-actin and expressed as fold change compared with WT control (defined as 1.0). *N* = 4–6 mice for each group (WT and PTENOE). Data are means ± SEM. ****p < 0.0001 versus WT. **C** Representative gel images showing doublecortin (DCX) in hippocampus. **D** Protein quantification data for hippocampus. Data are shown as mean ± SEM for each group. **** *p* < 0.0001 versus WT

Because elevated GPLD1 in plasma leads to higher BDNF and DCX in the brain [53], and because BDNF and DCS are both increased in the hippocampus of slow-aging mice [54–57, 64], we expected to find both proteins elevated in brain of PTENOE mice. Our data in Fig. 7 show that both proteins are indeed at significantly higher levels (1.6 to 2.2 × increase, *p* < 0.01) in PTENOE mice.

## Discussion

The importance of PTEN as a regulator of cell growth has been recognized and studied for many years. Its role in the PI3K/AKT pathway regulates cell division, in part by influence on function of mTOR. This relationship has been studied particularly with respect to cancer cell biology. More recent work has implicated PTEN as a factor in the biology of aging beyond its well-studied anti-cancer effects. Ortega-Molina has documented PTENOE effects on longevity, including increased insulin-sensitivity, reduced liver steatosis [6], increased lifespan independent of effects on cancer, increased energy expenditure, and low adiposity, and has shown increased UCP1 mRNA expression as an effect of hyperactive BAT [7]. We therefore sought to test the idea that PTEN overexpression might also alter multiple pathways that are frequently changed, together, in varieties of slow-aging mice.

Our previous research looked at nine varieties of slow-aging mice: Ames Dwarf, Snell Dwarf (*pou1f1* loss-of-function mutant), GH receptor knockout (GHRKO), and PAPPA knockout (PAPPA-KO); calorie-restricted (CR) mice; and mice treated with rapamycin (Rapa), acarbose (Aca), 17aE2, or canagliflozin (Cana). We described consistent changes in fat, fat-associated macrophages, muscle, liver, brain, and plasma. In some cases [65, 66], the physiological alterations induced by drugs with male-specific lifespan effects were noted in treated males only. The shifts caused by the CR diet and the drug treatments are seen in adults (typically 12 months of age). Specifically, they thus do not represent retardation of age-related changes — they are detectable in healthy young adults, and could potentially contribute to, rather than result from, retardation of aging and age-related changes in physiologic status. In that sense, they are not biomarkers of aging, but can instead be considered as indicators of aging rate, i.e., the pace of aging rather than the amount of prior aging. Many of the elements in this “aging rate indicator (ARI)” battery are already considered to be influential in disease processes that afflict mice and humans, such as the documented role of BDNF and DCX in preservation of cognitive function [67–69] and the role of inflammatory cytokines in metabolic health [70]. Our current work reinforces these links between slow aging and shared changes in ARIs, adding PTENOE to the set of long-lived mutants with ARI changes in multiple organs and cell types.

The set of proposed ARIs includes changes in six MAP kinases that mediate protein translation and inflammation [71], and specificity of mTORC1 function [72], which were not included in the present study of PTENOE mice. It will be of interest to evaluate these in future work. Members of our laboratory have also shown that both mutant and drug-treated slow-aging mice exhibit augmented cap-independent translation (CIT), leading to increases in a set of proteins independent of transcriptional changes [73]. GPLD1 is a member of this set [55], and the demonstration of higher GPLD1 levels in PTENOE mice suggests that proteome modification by selective mRNA translation may be characteristic of PTENOE mice as well as the other slow-aging mice.

It has been shown that elevation of PTEN levels in the mouse reduced body weight and size [3, 6]. Interestingly, the Garcia-Cao study examined multiple PTEN OE lines and found that the effect on body size was proportional to the level of PTEN expression [3]. The PTENOE mice used in our own work are smaller than control littermates, measured by body and femur length, and also have lower ING and PG WAT mass and higher BAT mass as a percentage of total body mass (Fig. 1). Dr. Garcia-Cao reported that IGF-1 levels were normal in his PTENOE mice [3]. However, we noted lower IGF1 levels in PTENOE mice, confirming previous results in another stock [6]; lower IGF1 seems likely to contribute to the smaller body size of mice. Lower plasma IGF1 is characteristic of other long-lived mouse models, such as Ames Dwarf, Snell Dwarf, GHRKO, and PAPPA-KO [74–77]. The reason for this decrease in the PTENOE mice is not known, and it is unclear if the effects of PTENOE on these ARIs are mediated by lower GH and/or IGF1 levels. However, we speculate that the role PTEN plays in the insulin/insulin-like growth factor signaling (IIS) pathway may be responsible for these positive effects on longevity and metabolic protection with age. The insulin/insulin-like growth factor (IGF)-1 signaling (IIS) pathway regulates aging in many species [78]. PTEN is a critical negative regulator of the IIS pathway through its role in the inhibition the PI3K reaction by its 3-phosphatase activity. It dephosphorylates PI(3,4,5)P_3_ converting it to PI(4,5)P_2_ [79, 80]. Many organisms have shown anti-aging responses to manipulations of this pathway from *Caenorhabditis elegans* to humans [81, 82]. The downregulation of mTOR and upregulation of FOXO are well-studied mechanisms that lead to longer lifespan [81]. Both of these results can be achieved by PTEN overexpression and the subsequent dephosphorylation of PIP3 in the PI3K/AKT pathway [5, 83, 84].

Age-related change in fat distribution, leading to a relative increase in the ratio of abdominal to subcutaneous fat mass, is blocked in several varieties of slow-aging mice [85]. Muzumdar et al. [86] demonstrated improved health and lifespan in rats after surgical removal of visceral fat, but not in rats that had a similar amount of subcutaneous fat removed. This result supports the idea that visceral fat (PG in our case) may have harmful effects not induced by equivalent mounts of subcutaneous fat [86]. Molina et al. found decreased epididymal WAT mass and BAT in 9-month-old PTENOE mice [6]. Our study confirms and expands upon this idea. Our data show a shift in relative mass of BAT and WAT, with declines in WAT (Ing and PG) accompanied by higher proportions of BAT in 4-month-old PTENOE mice. WAT and BAT differ widely in their functions: white adipose tissue is mainly used for long-term energy storage whereas brown adipose tissue has thermogenic properties [87]. The hallmark protein of brown adipose tissue’s increased energy production is UCP-1, which we found to increase in the BAT of PTENOE mice, consistent with Ortega-Molina’s observation of increased UCP1 mRNA expression [6]. Furthermore, we noted an increase in UCP1 in inguinal WAT of both sexes and in PG WAT of males. Ortega-Molina observed a similar pattern through histological staining that revealed an increase in mitochondria and brown adipose tissue in the WAT of the transgenic mice, a process known as fat “browning” [15]. This increased of brown fat mass has been consistently observed in the other long-lived mouse models we have worked with in the past (unpublished data) and is associated with increased energy expenditure as well as younger phenotypes in adipose tissue of older mice.

PTENOE mice also resemble previously studied slow-aging mice in the shift from M1 to M2 macrophages in WAT depots. PTENOE mice have previously been observed to have decreased insulin resistance and decreased liver steatosis [6], consistent with a syndrome of diminished inflammatory tone in WAT. M2 macrophages tend to secrete more anti-inflammatory and thermogenic cytokines [88, 89], which may contribute to the resistance to obesity in PTENOE mice. Our data suggest, however, that PG WAT of PTEN mice does not follow the pattern seen in other slow-aging mice. We observed the expected increase in Arg1 (M2) expression in male PG WAT, but found no significant change in female PG WAT. In addition, we did not see the expected decline in iNOS (M1) expression in PG WAT of males, and noted an unexpected increase in iNOS in PG WAT in female PTENOE mice. In most of our previous studies of long-lived mice, changes in M1 and M2 macrophages were seen in both inguinal and perigonadal WAT, as well as in BAT. We do not know why the perigonadal WAT of PTENOE mice follows a different, sex-specific pattern. There are notable differences between subcutaneous WAT (like ING WAT) and intra-abdominal WAT (like PG WAT) pertinent to aging. The two best-known are (a) a relative increase in subcutaneous WAT to intraabdominal WAT, as a function of age, in many kinds of slow-aging mice [85], and (b) lifespan extension [86] by surgical removal of intraabdominal fat, but not subcutaneous fat, in rats. We presume that PTEN may have a sex-specific effect on at least some intraabdominal fat depots that is independent of its effects on aging, but we do not know the molecular basis for depot-specific PTEN effects.

FNDC5 protein is induced by exercise, cleaved, and then secreted as irisin. Specifically, endurance exercise has been shown to increase FNDC5 levels in the hippocampi of mice [37, 90]. Lourenco et al. have further demonstrated the capacity of circulating FNDC5/irisin to enter the brain and elevate FNDC5/irisin expression while also providing protection against memory impairment [91]. Overexpression of irisin and FNDC5 was associated with neuroplasticity, neuronal proliferation, and neurotrophin synthesis [90, 92, 93]. Moreover, the increase in FNDC5 expression correlates positively with the expression of brain-derived neurotrophic factor (BDNF), one of the signaling molecules vital for synaptic plasticity and neurogenesis in the hippocampus [62]. Horowitz et al. showed that liver-secreted GPLD1 increased in the blood circulation of mice following exercise and that levels of the protein correlated closely with improvements in the animals’ cognitive performance [53]. Analysis of human data collected as part of the UCSF Memory and Aging Center’s Hillblom Aging Network study also found elevated blood levels in healthy, active, and elderly adults when compared to less active elders [94]. In summary, recent findings have revealed that FNDC5, irisin, and GPLD1 are important to maintaining the proper cognitive function of the nervous system.

In addition to the changes in adipose tissues, we observed higher levels of muscle FNDC5, plasma irisin, and liver and plasma GPLD1 in PTENOE mice. Exercise-induced FNDC5 and its cleavage product irisin have been linked to the increase in UCP1 and the subsequent WAT browning [36]. Increases in FNDC5 and irisin have also been seen to have neuroprotective benefits [95, 96]. This correlation between increased FNDC5/irisin and increased neurogenesis can be marked by the observed parallel increase in the two proteins DCX and BDNF in hippocampal tissue. In addition to the increase in irisin, we also found increased levels of GPLD1 in both the liver and plasma of PTENOE mice. GPLD1 has also been linked to increased cognitive function with age [53]. It is unclear to what extent GPLD1, locally synthesized irisin, and circulating irisin may interact to contribute to improvements in brain function and cognition.

PTEN is highly expressed in neurons [97, 98] and has been shown to regulate neuronal functions such as neurogenesis, neurite outgrowth, synaptogenesis, and synaptic plasticity [99, 100]. Our study showed that PTENOE mice have elevated hippocampal BDNF and DCX, consistent with our previous reports [54–57] on hippocampus in other slow-aging mice, and consistent with a report that increased GPLD1 in mice induces higher BDNF levels and improved cognitive function [53].

PTENOE mice have reduced MYC in multiple tissues [3], as do Snell and GHRKO mice [101]. Mice hemizygous for cMyc are long-lived, suggesting that lower levels of MYC protein may have beneficial health effects [102]. We report here that PTENOE mice share most of the suite of changes we have previously shown to characterize multiple forms of slow-aging mice. Many of the proteins that are altered in this set of 10 slow-aging mouse models contribute to diseases seen in old mice and which afflict older humans, thus providing potential links between the biology of aging and the pathophysiology of specific forms of late-life illness. Extension and refinement of this collection of ARIs may provide useful links to evaluation of putative anti-aging drugs in mice and in humans. Depending on the pace with which ARIs change after initiation of anti-aging drugs, a subset of ARIs might in principle be used to help screen candidate drugs to pick an agent more likely to have beneficial effects. Furthermore, greater insight into the processes by which each of the 10 mutants, drugs, and diets lead to coordinated changes in adipocyte biology, macrophage polarization, brain resiliency, and cap-independent translational control of proteome could suggest new, upstream, targets for pharmacological interventions that delay or retard multiple late-life problems.

### Supplementary Information

Below is the link to the electronic supplementary material.

Supplementary file1 (DOCX 16 KB)

Supplementary file2 (DOCX 16 KB)

Supplementary file3 (TIF 17114 KB)

Supplementary file4 (TIF 16272 KB)

## Data Availability

All raw images, densitometric data, and statistical calculations are available from the authors (XL, RAM) on request.
