# Supplementary material for: Recapitulation of anti-aging phenotypes by global overexpression of PTEN in mice
Source: GeroScience. 2023 Dec 19;46(2):2653–70. doi: 10.1007/s11357-023-01025-8 (PMC10828233; doi:10.1007/s11357-023-01025-8)
Supplement: Supplementary file 1 — Supplementary file1 (DOCX 16 KB) [file 11357_2023_1025_MOESM1_ESM.docx]

**Supplemental Table 1: Primer Sequences for Real-time PCR**

| Primer | Sequence | Primer | Sequence |
| --- | --- | --- | --- |
| mGAPDH-For | gacaactcactcaagattgtcagcaatgc | mGAPDH-For | gacaactcactcaagattgtcagcaatgc |
| mGAPDH-Rev | gtggcagtgatggcatggactgtggtc | mGAPDH-Rev | gtggcagtgatggcatggactgtggtc |
| UCP1-For | gggcccttgtaaacaacaaa | UCP1-For | gggcccttgtaaacaacaaa |
| UCP1-Rev | gtcggtccttccttggtgta | UCP1-Rev | gtcggtccttccttggtgta |
| Arg1-For | cagaacctgctgtcctgtga | Arg1-For | cagaacctgctgtcctgtga |
| Arg1-Rev | tgtcgttggaatcaacctga | Arg1-Rev | tgtcgttggaatcaacctga |
| iNOS-For | caccttggagttcacccagt | iNOS-For | caccttggagttcacccagt |
| iNOS-Rev | accactcgtacttgggatgc | iNOS-Rev | accactcgtacttgggatgc |

**Supplementary Table 2: Source of antibodies**

| **Antibody** | **Source** | **Cat #** |
| --- | --- | --- |
| **GPLD1** | Abcam | 210753 |
| **BDNF** | Abcam | 108318 |
| **Doublecortin** | Abcam | 18723 |
| **UCP1** | Abcam | 10983 |
| **Arg1** | Abcam | 124917 |
| **iNOS** | Abcam | 178945 |
| **FNDC5** | Abcam | 174833 |
| **Goat Anti-Rabbit IgG H&L (HRP)** | Abcam | 205718 |
| **PTEN** | Cell signaling | 9188s |
| **actin** | Abcam | 9644 |

| **Antibody** | **Source** | **Cat #** |
| --- | --- | --- |
| **GPLD1** | Abcam | 210753 |
| **BDNF** | Abcam | 108318 |
