# Supplementary material for: Recapitulation of anti-aging phenotypes by global overexpression of PTEN in mice
Source: GeroScience. 2023 Dec 19;46(2):2653–70. doi: 10.1007/s11357-023-01025-8 (PMC10828233; doi:10.1007/s11357-023-01025-8)
Supplement: Supplementary file 2 — Supplementary file2 (DOCX 16 KB) [file 11357_2023_1025_MOESM2_ESM.docx]

**Supplemental Table 3: Collection of statistical results of PTENOE**

| Tissue | Protein | Interaction  (PKO) | Sex  (PKO) | Genotype  (PKO) | Fold change |
| --- | --- | --- | --- | --- | --- |
| Inguinal fat | UCP1 | p = 0.48 | p = 0.66 | **p < 0.0001** | M: 1.7x, F: 1.4x |
| Perigonadal fat | UCP1 | **p < 0.001** | **p < 0.001** | p = 0.15 | M: 1.6x, F: 0.5x |
| BAT | UCP1 | p = 0.38 | p = 0.59 | **p < 0.001** | M: 1.4x, F: 1.4x |
| Inguinal fat | ARG1 | p = 0.08 | p = 0.1 | **p < 0.05** | M: 1.2x, F: 1.2x |
| Perigonadal fat | ARG1 | p = 0.2 | **p < 0.05** | p = 0.86 | M: 1.3x, F: 0.9x |
| BAT | ARG1 | p = 0.71 | p = 0.48 | **p < 0.05** | M: 1.4x, F: 1.4x |
| Inguinal fat | iNOS | p = 0.89 | p = 0.53 | **p < 0.05** | M: 0.6x, F: 0.6x |
| Perigonadal fat | iNOS | p = 0.65 | **p < 0.05** | p = 0.3 | M: 1.1x, F: 1.4x |
| BAT | iNOS | p = 0.29 | p = 0.85 | **p < 0.05** | M: 0.8x, F: 0.7x |
| Muscle | FNDC5 | p = 0.46 | p = 0.69 | **p < 0.05** | M: 1.2x, F: 1.1x |
| Hippocampus | FNDC5 | p = 0.95 | p = 0.89 | **p < 0.01** | M: 1.4x, F: 1.4x |
| Liver | GPLD1 | p = 0.20 | p = 0.84 | **p < 0.05** | M: 1.1x, F: 1.4x |
| Hippocampus | GPLD1 | p = 0.81 | p = 0.28 | p = 0.71 | M: 1.0x, F: 1.0x |
| Hippocampus | BDNF | p = 0.29 | p = 0.11 | **p < 0.0001** | M: 2.2x, F: 1.9x |
| Hippocampus | DCX | p = 0.19 | p = 0.51 | **p < 0.0001** | M: 2.2x, F: 1.6x |
